# Structural analysis of wild-type and Val120Thr mutant *Candida boidinii* formate dehydrogenase by X-ray crystallography

**DOI:** 10.1107/S2059798323008070

**Published:** 2023-10-20

**Authors:** Mehmet Gul, Busra Yuksel, Huri Bulut, Hasan DeMirci

**Affiliations:** a Department of Molecular Biology and Genetics, Koc University, 34450 Istanbul, Türkiye; b Max Planck Institute for Biophysics, 60438 Frankfurt am Main, Germany; c Department of Medical Biochemistry, Faculty of Medicine, Istinye University, 34010 Istanbul, Türkiye; d Koc University Isbank Center for Infectious Diseases (KUISCID), Koc University, 34010 Istanbul, Türkiye; e Stanford PULSE Institute, SLAC National Laboratory, Menlo Park, CA 94025, USA; University of Konstanz, Germany

**Keywords:** formate dehydrogenases, *Candida boidinii*, protein engineering, X-ray crystallography, structural biology, structural dynamics, Turkish Light Source, Turkish DeLight

## Abstract

*Candida boidinii* NAD^+^-dependent formate dehydrogenase (*Cb*FDH) has gained significant attention for its potential application in the production of biofuels and various industrial chemicals from inorganic carbon dioxide. The present study reports the atomic X-ray crystal structures of wild-type *Cb*FDH at cryogenic and ambient temperatures, as well as that of the Val120Thr mutant at cryogenic temperature, determined at the Turkish Light Source ‘Turkish DeLight’. The structures reveal new hydrogen bonds between Thr120 and water molecules in the active site of the mutant *Cb*FDH, suggesting increased stability of the active site and more efficient electron transfer during the reaction. Further experimental data is needed to test these hypotheses. Collectively, these findings provide invaluable insights into future protein-engineering efforts that could potentially enhance the efficiency and effectiveness of *Cb*FDH.

## Abstract 2

This study presents the atomic X-ray crystal structures of wild-type *Candida boidinii* NAD^+^-dependent formate dehydrogenase (*Cb*FDH) and its Val120Thr mutant, revealing new hydrogen bonds and increased stability in the active site of the mutant. These findings offer valuable insights for protein engineering, potentially improving the efficiency and electron transfer of *Cb*FDH for applications in biofuel production and industrial chemical synthesis from carbon dioxide.

## 1. Introduction

*Candida boidinii* NAD^+^-dependent formate dehydrogenase (*Cb*FDH) is a homodimeric 82 kDa enzyme complex that catalyzes the reversible conversion of formate and carbon dioxide (Nielsen *et al.*, 2019 ▸) using NAD^+^/NADH coenzymes for electron transfer (Niks & Hille, 2018 ▸). Many prokaryotic and eukaryotic organisms express NAD^+^-dependent formate dehydrogenase enzymes with similar structural and kinetic properties (Popov & Lamzin, 1994 ▸). However, many studies have focused on *Cb*FDH due to its remarkable stability and activity (Bulut *et al.*, 2021 ▸).

Formate dehydrogenase (FDH) was first discovered in the 1950s and has recently gained great attention as a research topic due to its potential for the production of formate molecules from inorganic carbon dioxide. Formate is a valuable precursor molecule that is used in the synthesis of industrial chemicals such as formaldehyde, which has numerous applications in the production of resins, plastics and disinfectants (Aronson, 2016 ▸; Murashima *et al.*, 2022 ▸; Powers, 1953 ▸). Other chemicals that can be generated from formate include formic acid and oxalic acid, both of which have a range of important industrial applications (Schuler *et al.*, 2021 ▸). Moreover, the formate molecule has potential as an alternative biofuel since it can be converted into hydrogen gas by electrolysis (Eppinger & Huang, 2017 ▸). Overall, the versatility of formate in industrial applications has made the FDH enzyme a central research topic.

FDH has also emerged as a highly promising candidate for carbon-capture efforts to counteract global climate change. The inherent capability of the enzyme to catalyze the conversion of CO_2_ to formate holds significant potential for reducing atmospheric CO_2_ levels (Calzadiaz-Ramirez & Meyer, 2022 ▸; Choe *et al.*, 2014 ▸; Miller *et al.*, 2019 ▸). Nonetheless, to optimize its efficiency and robustness in CO_2_ conversion, studies that focus on engineering FDH are necessary. These involve increasing its thermostability to endure harsh conditions and enhancing the enzymatic activity for the reverse reaction, making it an even more suitable option for carbon-capture strategies (Calzadiaz-Ramirez & Meyer, 2022 ▸). In order to enhance stability and activity, researchers have made significant progress by identifying and using enzymes from organisms that live in extreme environments. While *C. boidinii* might not be considered to be an extremophile, these studies have shown the potential of extremophiles as valuable genetic resources. Extremophiles have provided insight into ways to make enzymes stable in different industrial and environmental situations. The stability-enhancing adaptations observed in extremophilic enzymes may inspire protein engineers to employ similar strategies to improve enzymes such as *Cb*FDH, even when the host organism is not an extremophile (Chen & Jiang, 2018 ▸; Dumorné *et al.*, 2017 ▸; Ye *et al.*, 2023 ▸).

The industrial and therapeutic applications of proteins are significantly impacted by their limited activity and stability under various industrial conditions. Therefore, protein-engineering studies are crucial to generate mutant proteins to overcome these limitations and improve protein function. Protein-engineering studies with the help of computational biology tools allow us to identify and mutate amino-acid residues that may improve the thermostability of proteins. Thermostable mutants are suitable for industrial biocatalysis due to their increased resistance to high temperatures. These mutations include single or multiple amino-acid alterations, insertions or deletions to give greater numbers of hydrogen bonds, disulfide bonds or salt bridges and increase the thermostability (Modarres *et al.*, 2016 ▸). In addition, protein-engineering approaches are also focused on creating proteins with higher enzymatic activity. More active mutant proteins can increase the rate and efficiency of the reaction. Similar to stability, protein-engineering studies are used to identify and modify amino acids that can improve activity, specificity and substrate affinity. Site-directed mutagenesis, random mutagenesis and DNA-shuffling methods are widely used to generate highly active mutant proteins (Li *et al.*, 2020 ▸). In *Cb*FDH, residue 120 (Val120) is a potential mutation site for studies aiming to create a more active enzyme variant as it is located in the catalytic and substrate-binding site (Supplementary Fig. S1). Although it is unclear whether Val120 is directly involved in the reaction, mutating it to a more polar residue such as serine results in enhanced catalytic activity. This might be due to changes in charge distribution within the active site of the enzyme (Jiang *et al.*, 2016 ▸).

High-resolution structural studies are crucial for understanding the function, the underpinnings of the catalytic mechanism and other potential applications of dehydrogenase enzymes such as *Cb*FDH. Previous studies have contributed to our knowledge of the structural and functional features of *Cb*FDH. However, there are still gaps in our understanding of its function and potential modifications to further enhance its activity and thermal stability. Here, we present high-resolution crystal structures of apo *Cb*FDH determined at cryogenic and ambient temperatures, as well as as that of the highly active Val120Thr mutant of *Cb*FDH at cryogenic temperature. Our findings, combined with existing knowledge of the structure and function of the enzyme, will drive further research on the potential industrial applications of *Cb*FDH.

## 2. Materials and methods

### 2.1. Transformation, expression, purification and crystallization

Full-length wild-type and Val120Thr mutant *Cb*FDH genes were cloned into pET-23a(+) bacterial expression vector with a C-terminal hexahistidine tag as described previously by Bulut *et al.* (2021 ▸). The plasmids were transformed into the competent *Escherichia coli* BL21 Rosetta 2 strain. Transformed bacterial cells were grown in 6 l LB medium containing 100 µg ml^−1^ ampicillin and 35 µg ml^−1^ chloramphenicol at 37°C. Once OD_600_ reached the range 0.7–0.8, protein expression was induced by the addition of isopropyl β-d-1-thiogalactopyranoside (IPTG) to a final concentration of 0.4 m*M* for 3–4 h at 37°C. The cells were then harvested using a Beckman Allegra 15R desktop centrifuge at 4°C at 3500 rev min^−1^ for 30 min. The cell pellets were stored at −80°C until purification.

The frozen cell pellets were dissolved in ice-cold lysis buffer consisting of 500 m*M* NaCl, 50 m*M* Tris–HCl pH 7.5, 5%(*v*/*v*) glycerol, 0.1%(*v*/*v*) Triton X-100 and sonicated on ice with a Branson W250 sonifier (Brookfield, Connecticut, USA) until the solution was completely cleared. The cell lysate was centrifuged at 4°C at 35 000 rev min^−1^ for 1 h using a Beckman Optima L-80XP ultracentrifuge equipped with a Ti-45 rotor (Beckman, USA). The cell pellet was discarded and the supernatant containing the soluble protein was filtered through a 0.2 µm membrane and loaded onto an Ni–NTA column that had previously been equilibrated with three column volumes of loading buffer consisting of 200 m*M* NaCl, 20 m*M* Tris–HCl pH 7.5, 20 m*M* imidazole. Unbound and non­specifically bound proteins were washed by running five column volumes of loading buffer through the column. The hexahistidine-tagged *Cb*FDH proteins were eluted with elution buffer consisting of 200 m*M* NaCl, 20 m*M* Tris–HCl pH 7.5, 250 m*M* imidazole. The eluted proteins were dialyzed in a dialysis membrane (3 kDa molecular-weight cutoff) against a buffer consisting of 200 m*M* NaCl, 20 m*M* Tris–HCl pH 7.5 overnight at 4°C to remove excess imidazole. The concentrated pure proteins were run on 15% SDS–PAGE for verification.

Protein crystallization screening experiments were performed using the sitting-drop microbatch-under-oil method. The purified wild-type and Val120Thr mutant *Cb*FDH protein were mixed with ∼3500 commercially available sparse-matrix crystallization screening conditions in a 1:1 volumetric ratio in 72-well Terasaki plates (Greiner Bio-One, Kremsmünster, Austria). The mixtures were covered with 16.6 µl 100% paraffin oil (Tekkim Kimya, Istanbul, Türkiye). The crystallization plates were incubated at 4°C and frequently checked under a light microscope. The best *Cb*FDH crystals grew within six weeks in Wizard III condition No. 47 (Hampton Research, USA) for both wild-type and mutant protein. This condition consists of 30%(*w*/*v*) PEG 5000 MME, 100 m*M* MES–sodium hydroxide pH 6.5, 200 m*M* ammonium sulfate.

### 2.2. Activity assays for enzyme kinetics

Activity assays were performed for wild-type and mutant *Cb*FDH at different substrate, coenzyme and enzyme concentrations in order to calculate the *K*_m_, *V*_max_ and *k*_cat_ values. The values were used to plot Michaelis–Menten and Lineweaver–Burk graphs, which yielded an equation for calculations. The reaction conditions used for measurements are shown in Table 1 ▸.

The measurements were performed at 25°C and 340 nm in a microplate reader (Varioscan, ThermoFisher Scientific, USA).

### 2.3. Crystal harvesting and delivery

The *Cb*FDH crystals were harvested using MiTeGen MicroLoops mounted on a magnetic wand (Garman & Owen, 2006 ▸) with simultaneous monitoring under a light microscope as described by Atalay *et al.* (2023 ▸). The harvested crystals were flash-cooled by plunging them into liquid nitrogen and were placed in a cryocooled robotic sample puck (catalog No. M-CP-111-021, MiTeGen, USA). The puck was mounted onto a transfer and mounting tool and was placed into the sample dewar, which is auto-refilled with liquid nitrogen at 100 K, at the Turkish DeLight source (Gul *et al.*, 2023 ▸).

### 2.4. Data collection and processing

Diffraction data were collected from the *Cb*FDH crystals using a Rigaku XtaLAB Synergy Flow X-ray diffractometer at the University of Health Sciences, Istanbul, Türkiye controlled with the *CrysAlis^Pro^* software (version 1.171.42.35a; Rigaku Oxford Diffraction). The crystals were constantly cooled by an Oxford Cryosystems Cryostream 800 Plus set to 100 K. The PhotonJet-R X-ray generator was operated at 40 kV, 30 mA and 1200.0 W with a beam intensity of 10%. The data were collected at a wavelength of 1.54 Å and the detector distance was 47.00 mm. The scan width was set to 0.25°, while the exposure time was 10.0 min. Data collection was performed for 15 h 43 min 19 s.

Data collection at ambient temperature was performed as described by Gul *et al.* (2023 ▸). Data reduction was performed using *CrysAlis^Pro^* version 1.171.42.35a. The data-reduction result was obtained as an *.mtz file.

### 2.5. Structure determination and refinement

The cryogenic *Cb*FDH structure was determined at 1.4 Å resolution in space group *P*1 using the automated molecular-replacement program *Phaser* (version 2.8.3; McCoy *et al.*, 2007 ▸) within the *Phenix* suite (version 1.20.1; Liebschner *et al.*, 2019 ▸). The previously released *Cb*FDH structure with PDB code 5dna (Guo *et al.*, 2016 ▸) was used as the initial search model for molecular replacement of the wild-type *Cb*FDH structures. For Val120Thr-*Cb*FDH, Val120 of the wild-type *Cb*FDH structure was mutated to threonine in *PyMoL* for use as the search model in molecular replacement. Initially, rigid-body and simulated-annealing refinement were performed in *Phenix*. Individual coordinates and translation/libration/screw (TLS) parameters were then refined along with simulated-annealing map refinement. The final model of the *Cb*FDH structure was checked using *Coot* version 0.9.8.1 (Emsley *et al.*, 2010 ▸) after each round of refinement. Water molecules were added into appropriate electron-density clouds, while those located outside the density were manually removed. The structures were refined until *R*_work_ and *R*_free_ were sufficiently low (Brünger, 1992 ▸). Structural figures were generated using *PyMOL* (version 2.5.2; Schrödinger) and *Coot* (version 0.9.8.1).

## 3. Results

### 3.1. Wild-type apo *Cb*FDH structure determined at 1.4 Å resolution at cryogenic temperature

**Figure 1:**
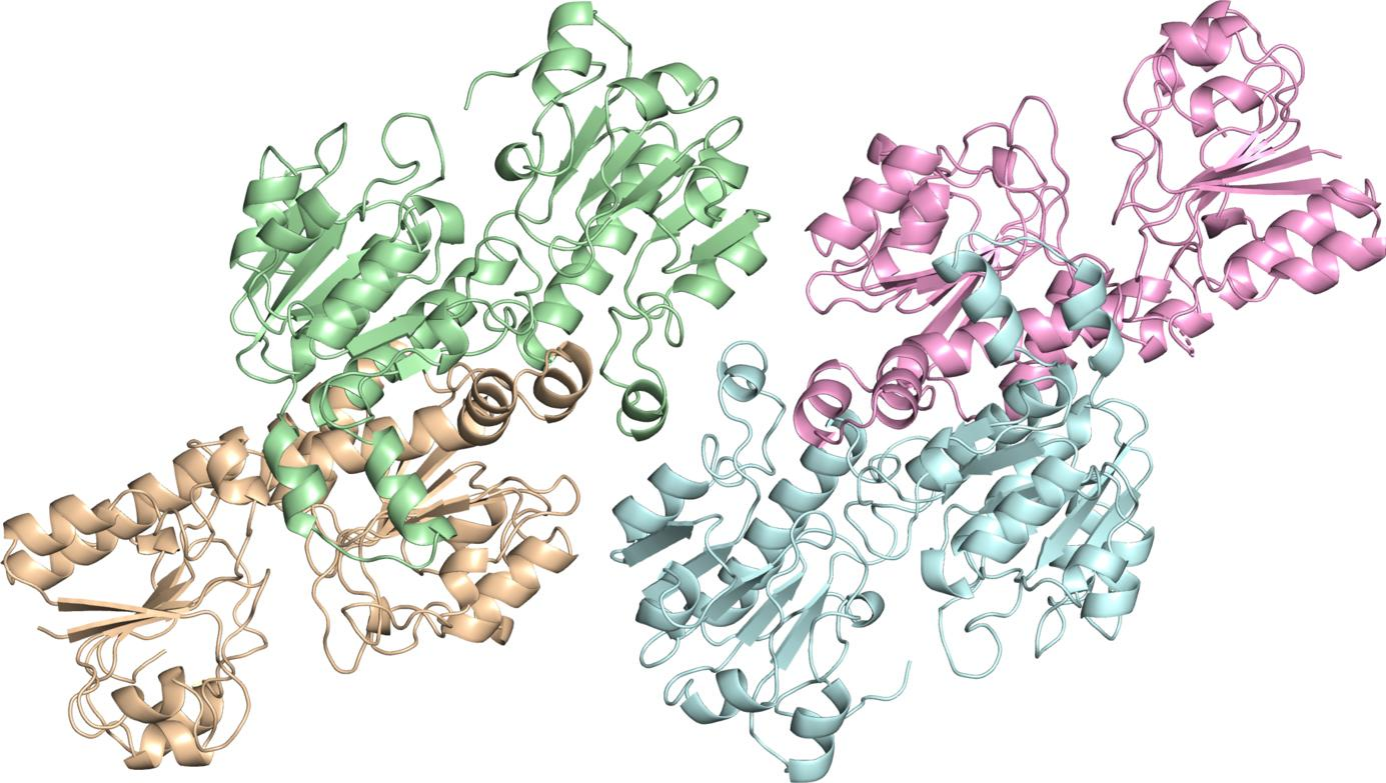
The 1.4 Å resolution apo wild-type *Cb*FDH structure comprising two homodimers. Four monomer molecules are shown in different colors. The monomers in pale green and wheat form the first dimer, while the monomers in pink and pale cyan form the second dimer.

We determined the crystal structure of wild-type apo *Cb*FDH at 1.4 Å resolution (PDB entry 8hty) with an overall completeness of 98.82% at cryogenic temperature at the Turkish Light Source (‘Turkish DeLight’), Istanbul, Türkiye. The determined structure consists of two homodimers of *Cb*FDH (Fig. 1 ▸). The Ramachandran plot indicates that 98.01% of the residues were in favored regions, while 1.99% were in allowed regions, with no outliers. The data-collection and refinement statistics are given in Table 2 ▸.

**Figure 2:**
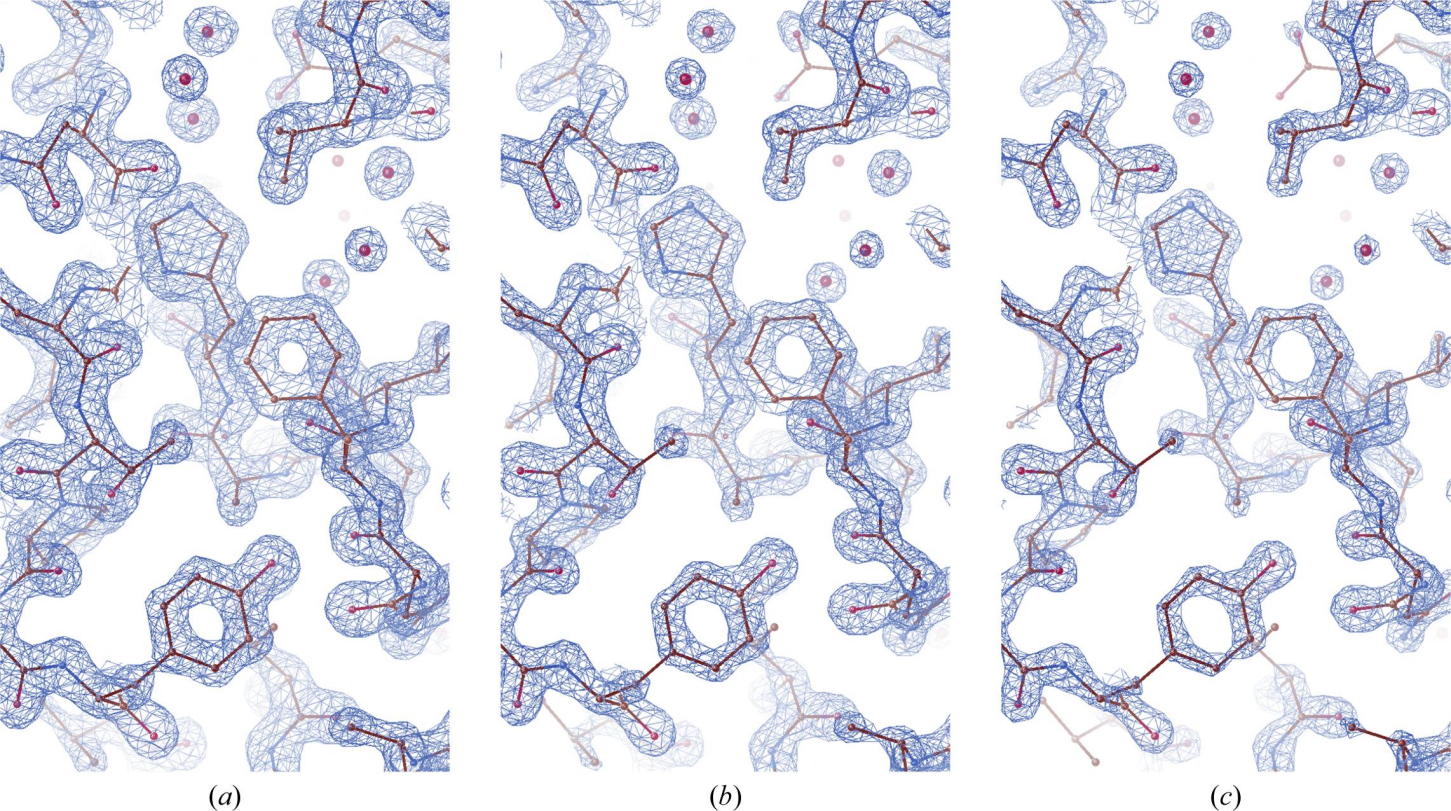
The 2*F*_o_ − *F*_c_ map is colored blue and contoured at (*a*) 2.0σ, (*b*) 2.5σ and (*c*) 3.0σ levels. *Cb*FDH is shown in stick representation.

The resulting final electron-density map was of superior quality and revealed structural features such as coordinated water molecules and side chains of the *Cb*FDH molecule in great detail. The electron density remains intact and visible at the 3σ level (Fig. 2 ▸).

We superposed the two *Cb*FDH homodimers in our crystal structure to compare their similarities (Supplementary Fig. S2). The two homodimers aligned with an r.m.s.d. of 0.352 Å. In particular, the dimerization regions within the *Cb*FDH homodimers appeared to be almost identical (Supplementary Fig. S2*c*), while some minor conformational changes are observed in regions farther from the central dimerization core domain (Supplementary Figs. S2*b* and S2*d*).

### 3.2. Wild-type apo *Cb*FDH structures align with some minor differences

**Figure 3:**
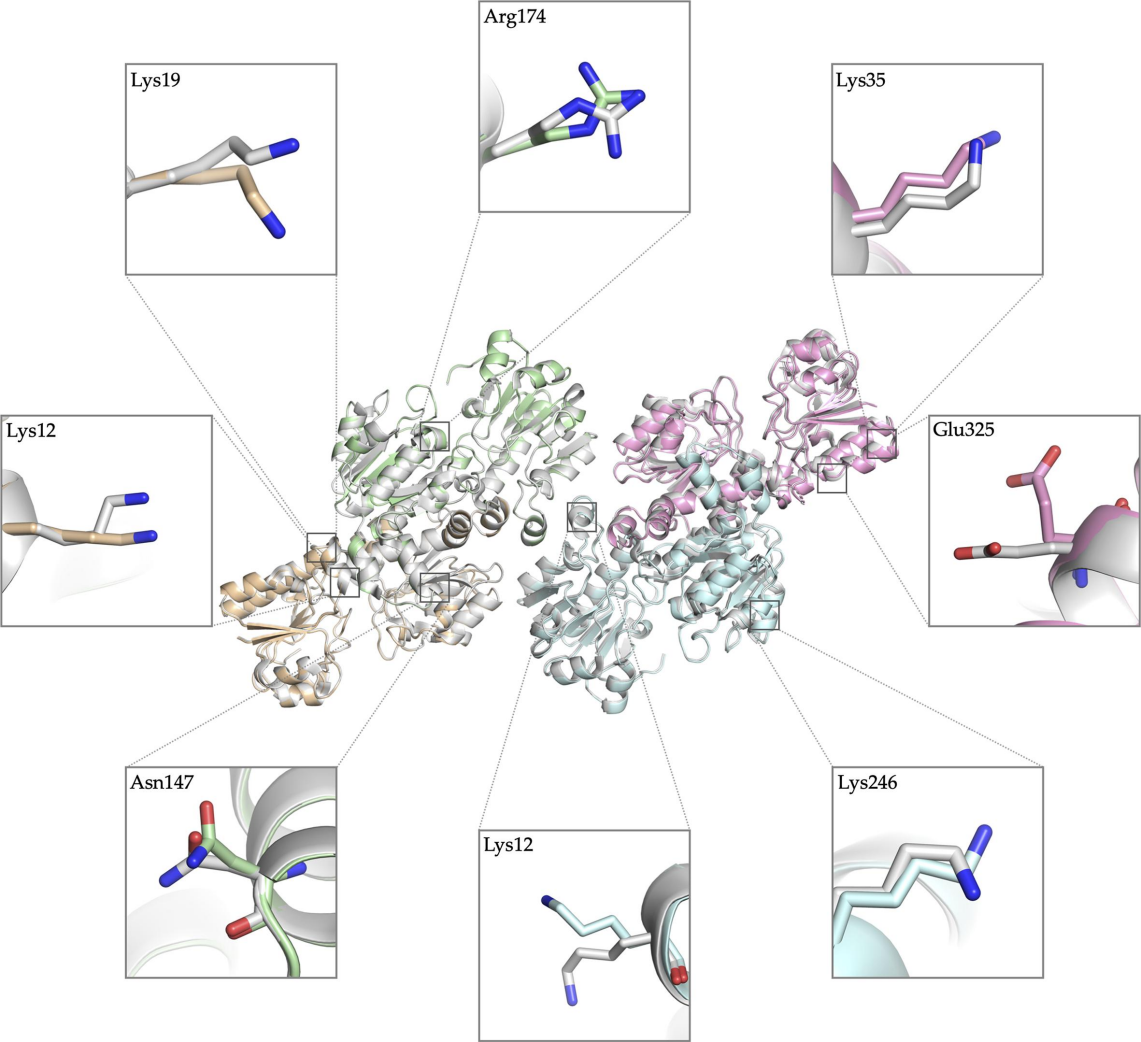
Superposition of two apo wild-type *Cb*FDH structures. Our 1.4 Å resolution structure is shown in four distinct colors indicating each chain: *A*, pale green; *B*, wheat; *C*, pink; *D*, pale cyan. PDB entry 5dna is colored gray. Conformational differences in side chains are shown in boxes in stick representation.

Our wild-type apo *Cb*FDH structure crystallized in the same space group and unit cell as the previous structure (PDB entry 5dna; Guo *et al.*, 2016 ▸). When we compare our 1.4 Å resolution apo *Cb*FDH structure with the previously published apo structure (PDB entry 5dna) at 1.75 Å resolution the overall structures align very well, with an r.m.s.d. of 0.266 Å, as expected (Fig. 3 ▸).

Differences are mostly observed in residues that are directly exposed to the solvent (Fig. 3 ▸). In addition, the previous apo *Cb*FDH structure lacks residues 15–18 (Ala15, Asp16, Glu17 and Glu18), which are part of a loop in chain *C*, while our structure has well defined electron density for these residues (Supplementary Fig. S3).

### 3.3. Wild-type apo *Cb*FDH structure determined at 2.1 Å resolution at ambient temperature

**Figure 4:**
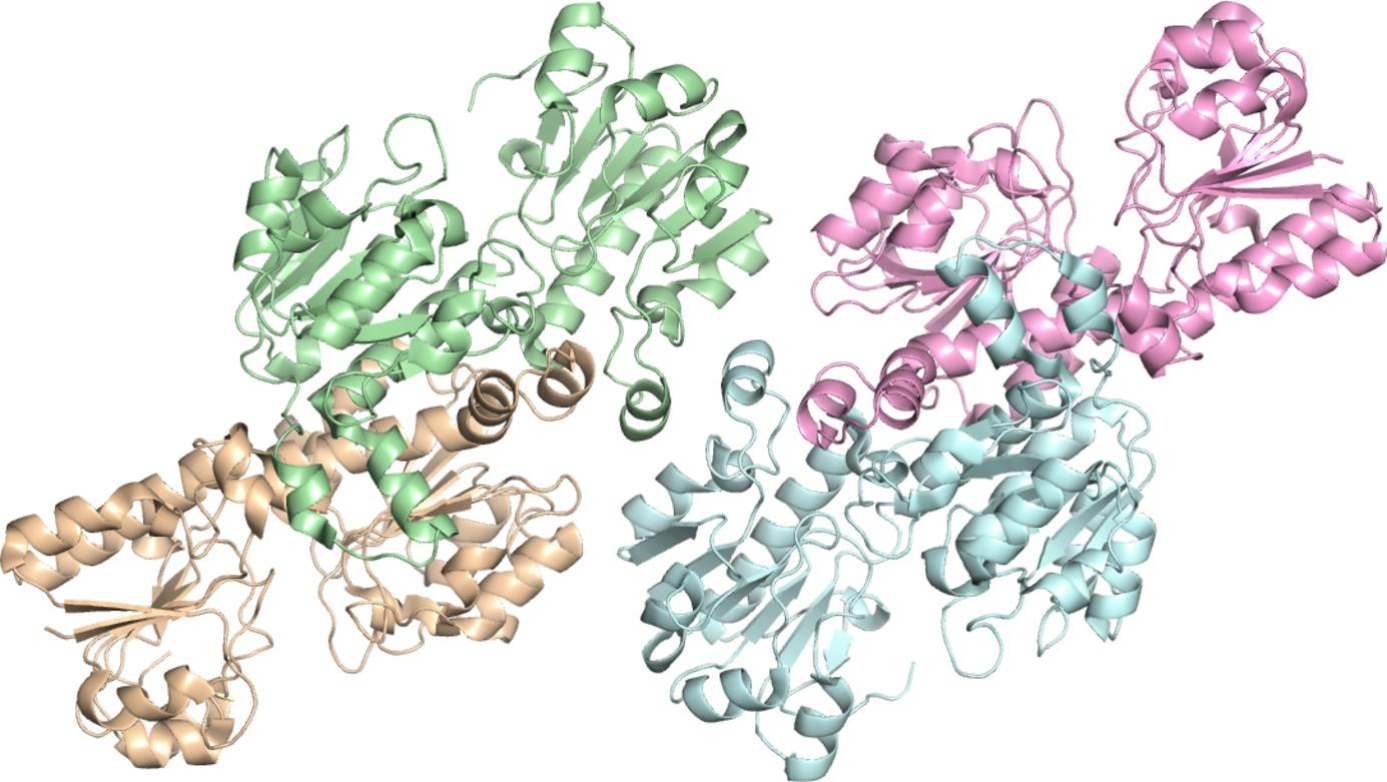
Wild-type apo *Cb*FDH structure at 2.1 Å resolution at ambient temperature.

The crystal structure of wild-type apo *Cb*FDH was determined at at the Turkish Light Source (‘Turkish DeLight’), Istanbul, Türkiye at 2.1 Å resolution (PDB entry 8iq7) with 85.9% completeness at ambient temperature. Similar to the cryogenic structure, the structure consists of two homodimers (Fig. 4 ▸). 97.66% of the residues were in the favored regions, while 2.34% were in the allowed regions, with no outliers according to the Ramachandran statistics. The data-collection and refinement statistics are given in Table 2 ▸.

### 3.4. Cryogenic and ambient apo *Cb*FDH structures possess slight differences

**Figure 5:**
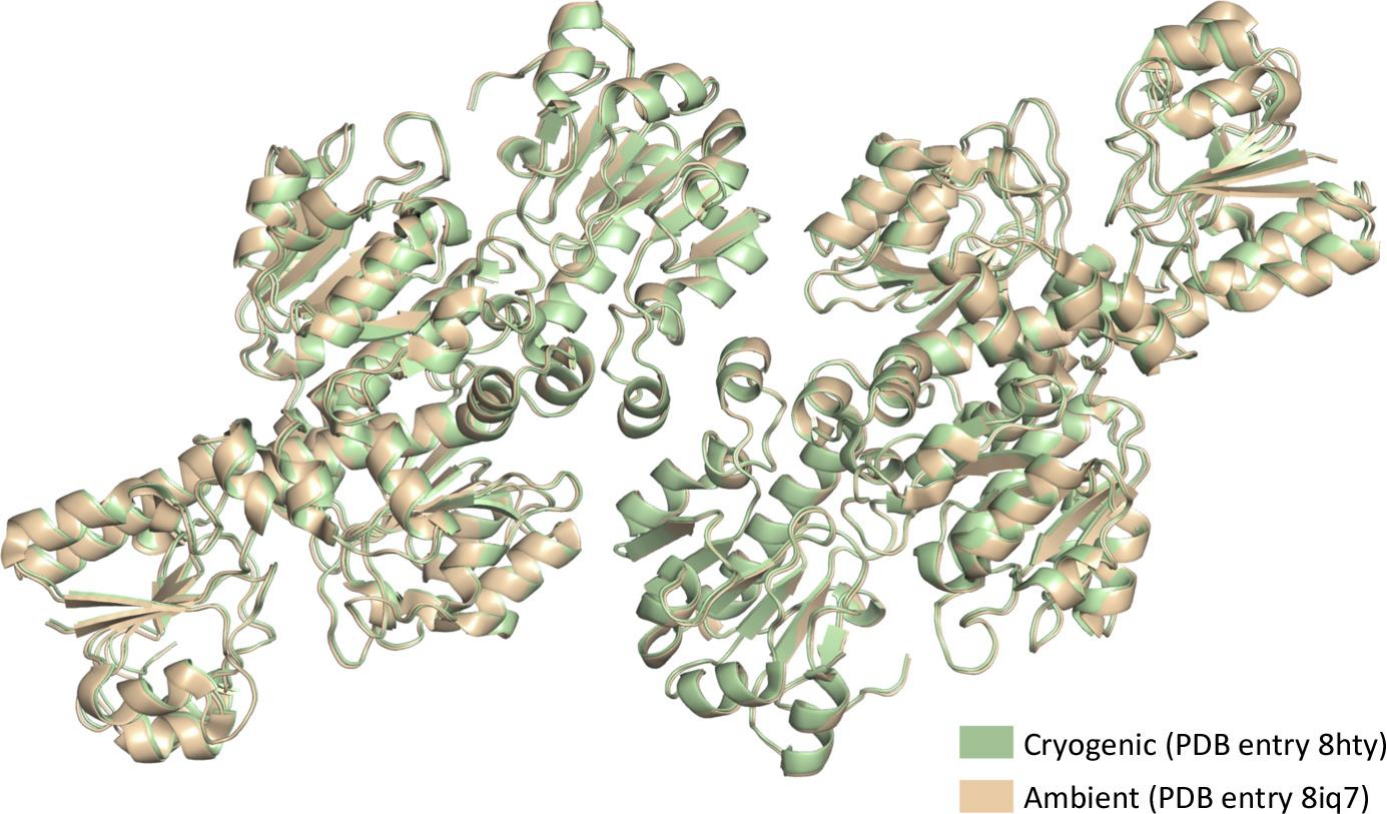
Superposition of cryogenic and ambient wild-type *Cb*FDH structures.

Our cryogenic (PDB entry 8hty) and ambient (PDB entry 8iq7) *Cb*FDH structures superpose with an r.m.s.d. of 0.554, suggesting slight differences between the two structures. These differences are mostly in the flexible-loop regions and amino-acid side chains (Fig. 5 ▸).

We also compared the number of water molecules identified within our cryogenic and ambient temperature structures. The cryogenic temperature structure exhibited a significantly higher number of identified water molecules, with 2179 waters resolved, compared with the ambient temperature structure with only 393 water molecules.

### 3.5. Mutation of valine to threonine significantly enhances the activity of *Cb*FDH

Kinetic assays were performed to assess the changes in *Cb*FDH activity due to the mutation of valine to threonine at residue 120. The *K*_m_, *k*_cat_ and *k*_cat_/*K*_m_ values calculated for formate are shown in Table 3 ▸. The lower *K*_m_ value for formate for the Val120Thr mutant indicates an approximately 1.5-fold stronger affinity for substrate binding, while the higher *k*_cat_ indicates a higher turnover number (6.57-fold) compared with the wild type. The overall catalytic efficiency (*k*_cat_/*K*_m_) is found to be approximately ten times greater in the mutant *Cb*FDH.

### 3.6. The *Cb*FDH Val120Thr mutant structure reveals new hydrogen bonds in the active site

We determined the structure of the novel Val120Thr mutant of *Cb*FDH at 1.9 Å resolution at cryogenic temperature (PDB entry 8ivj). The structure is composed of two homodimers with 99.7% completeness. The Ramachandran statistics indicate that 98.02% of the residues are in favored regions and 1.98% are in allowed regions, with no outliers. Table 2 ▸ shows the data-collection and refinement statistics.

**Figure 6:**
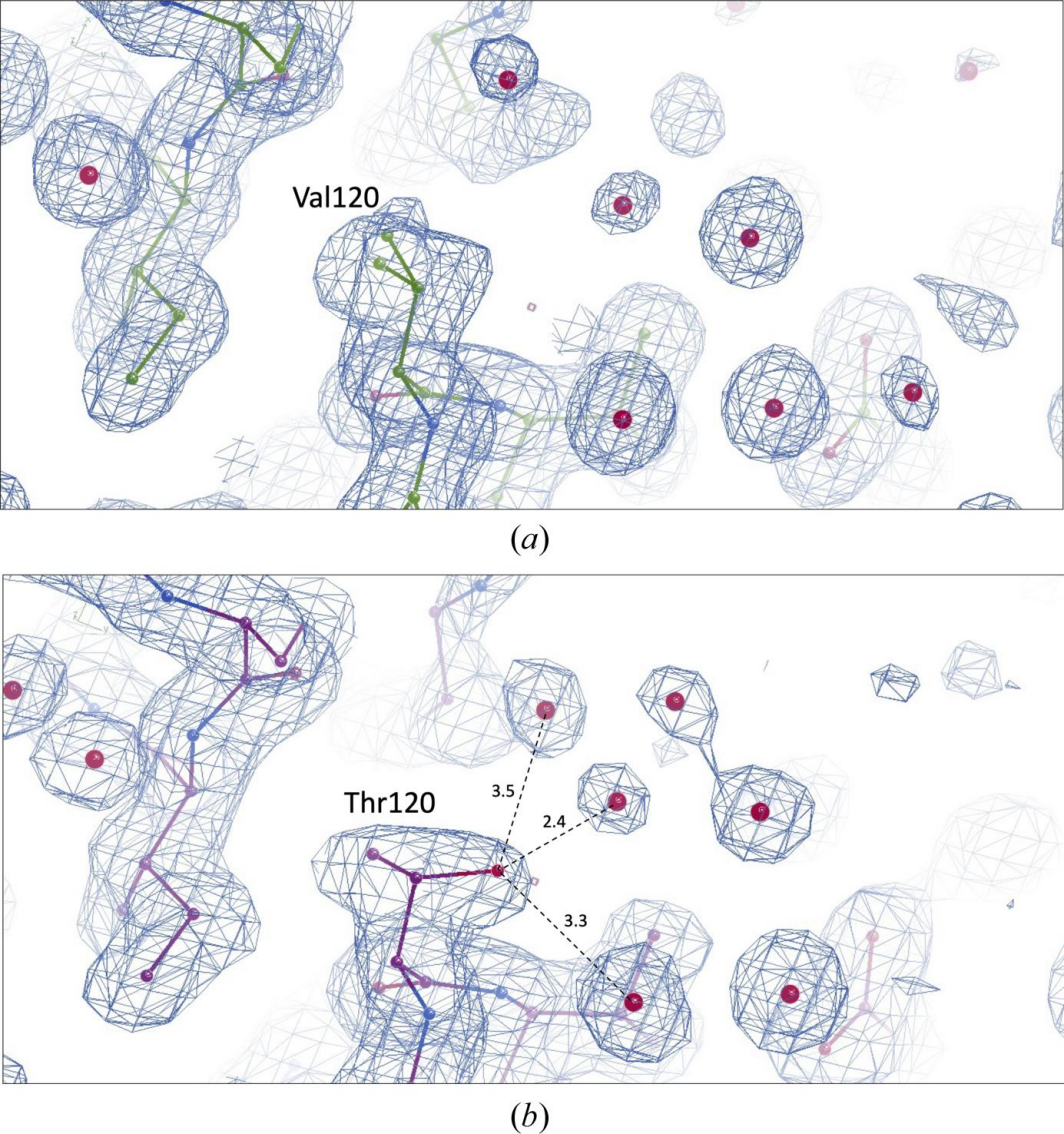
2*F*_o_ − *F*_c_ simulated-annealing omit map of residue 120 of *Cb*FDH, shown in blue and contoured at the 1.0σ level (Brünger *et al.*, 1998 ▸; Hodel *et al.*, 1992 ▸). (*a*) Val120 of the cryogenic wild-type *Cb*FDH structure (PDB entry 8hty). (*b*) Thr120 of the cryogenic Val120Thr mutant *Cb*FDH (PDB entry 8ivj). Hydrogen bonds are indicated by dashed lines with distances in Å.

Superposition of wild-type *Cb*FDH and the Val120Thr mutant (Supplementary Fig. S4*a*) demonstrated that the overall structure does not change significantly as a consequence of this single mutation (r.m.s.d. of 0.270 Å). However, a closer look at the mutation site revealed new hydrogen bonds that does not exist in wild-type *Cb*FDH between the threonine at position 120 and three water molecules (Fig. 6 ▸; Supplementary Figs. S4*b*–S4*f*).

## 4. Discussion

Our 1.4 Å resolution apo *Cb*FDH structure provides structural details of the enzyme in its homodimeric form. The superior-quality electron-density map reveals atomic details of the amino-acid residues and water molecules. This high-resolution structure revealed a high degree of similarity between the two subunits, with only minor differences being observed in the more flexible regions outside the dimerization core domain region. These minor changes are expected since these regions can move more freely as they do not participate in dimer formation.

While the two homodimers in our structure were highly identical, comparison with a previously published apo structure, also consisting of two homodimers (PDB entry 5dna), at 1.75 Å resolution revealed several minor differences, particularly in the side chains of solvent-exposed charged residues. Furthermore, our structure also showed improved electron density for flexible-loop residues 15–18 (Ala15, Asp16, Glu17 and Glu18) in chain *C* that were not well defined in the previous structure.

Studying protein structures at ambient temperature offers valuable insights into their natural conformational dynamics, active-site flexibility, functional mechanisms and binding behavior. This approach may capture a broader range of conformational states, especially for proteins that are sensitive to cryogenic temperatures (Fischer, 2021 ▸; Fraser *et al.*, 2011 ▸). By combining insights from both cryogenic and ambient temperature structures, we may gain a more complete understanding of the structures of proteins and their dynamics. Therefore, in addition to the structure obtained at cryogenic temperature, we also determined the structure of wild-type *Cb*FDH at ambient temperature. Comparison of the wild-type structures determined at cryogenic and ambient temperatures showed that the structure did not significantly change due to temperature except for flexible loops and large amino-acid side chains. The reason might be that flash-cooling in liquid nitrogen captured the protein in its most stable state in solution, which we also observed in the ambient temperature structure. Still, the main difference between the two structures is most likely to be in flexibility or mobility. However, the difference in resolution may affect comparison of the *B* factors. Thus, further analysis is required to fully understand the impact of temperature on the structure.

The significant difference in the number of water molecules identified within the cryogenic and ambient temperature structures raises intriguing questions about the dynamic interactions between protein and water under different environmental conditions. The higher resolution achieved at cryogenic temperatures significantly improves the accuracy of identifying water molecules compared with the ambient temperature structures at lower resolution. Another reason for the different numbers of identified water molecules could be the differences in water accessibility and occupancy between the two conditions. At cryogenic temperatures water molecules might be frozen at specific binding sites, leading to the stabilization of interactions that are transient at ambient temperature (Nakasako, 2004 ▸). This could explain why cryogenic temperature structures allow the identification of more water molecules compared with ambient temperature structures. These findings emphasize the importance of investigating temperature effects as the microenvironment of the protein exhibits high complexity, with changes in conformation and water interactions due to environmental factors. Further studies, such as molecular-dynamics simulations and spectroscopic techniques, can provide further insights into protein–water interactions and water-molecule behavior.

According to the results of the kinetic assay, the mutant *Cb*FDH has a lower *K*_m_ value, indicating a higher affinity for formate, and a higher *k*_cat_ value, indicating a faster conversion to the product. Overall, the catalytic efficiency is increased approximately tenfold compared with the wild-type enzyme. The reason for the increase in substrate affinity may be that threonine is more polar than valine, so that it can form hydrogen bonds to neighboring amino acids or water molecules, resulting in higher stability in the substrate-binding region. In addition, it may allow easier substrate binding by providing a larger space for formate to enter as threonine has a smaller side chain compared with valine.

In order to observe the effect of the Val120Thr mutation, we determined the structure of the mutant *Cb*FDH at 1.9 Å resolution and compared it with our wild-type structure. When the structures were superimposed, we did not observe major changes in the structure, as expected. In the mutation site, nonetheless, we observed three new hydrogen bonds between Thr120 and water molecules in the active site. These new hydrogen bonds may help to stabilize the active site, leading to greater activity. Also, the newly formed hydrogen bonds may help to position the substrate or coenzyme molecules in the active site and contribute to a more efficient electron-transfer process during the reaction. Additionally, the hydroxyl group of the threonine side chain could affect the binding of the NAD^+^ coenzyme by interacting with it or other amino acids in the active site. In order to test these hypotheses, further experimental data is needed, including co-crystallization of the Val120Thr mutant *Cb*FDH with NAD^+^ and formate/azide.

The high-resolution structures of apo *Cb*FDH presented in this study provide insight into the structural details of the enzyme. Moreover, the Val120Thr mutation in the active site of the enzyme enhanced the enzymatic activity approximately tenfold compared with the wild-type enzyme. The structural changes observed between wild-type and mutant *Cb*FDH emphasize the significance of structural analysis in understanding the effects of mutations on enzyme activity. Further research on the mutant enzyme in complex with its substrate and coenzyme is required to fully understand the underlying mechanism of the mutation. Overall, the findings in this study can potentially contribute to the development of enzymes with higher efficiency and specificity for a range of applications in biotechnology, industry and medicine.

## 5. Data availability

The cryogenic wild-type, ambient temperature wild-type and cryogenic Val120Thr mutant *Cb*FDH structures presented in this manuscript have been deposited in the Protein Data Bank under accession codes 8hty, 8iq7 and 8ivj, respectively. Any remaining information can be obtained from the corresponding author upon request.

## Supplementary Material

PDB reference: *Candida boidinii* formate dehydrogenase, cryogenic temperature, 8hty

PDB reference: ambient temperature, 8iq7

PDB reference: V120T mutant, 8ivj

Supplementary Figures. DOI: 10.1107/S2059798323008070/di5067sup1.pdf

## Figures and Tables

**Table 1:** Reaction conditions for activity assays

Condition	Concentrations
Formate concentration (m*M*)	0.612, 1.25, 2.50, 5.00, 10.00, 20.00, 40.00
NAD^+^ concentration (m*M*)	1.00, 2.00, 3.00, 4.00, 5.00, 6.00
*Cb*FDH concentration (mg ml^−1^)	0.0306, 0.0612, 0.125, 0.25, 0.50, 0.75

**Table 2:** Data-collection and refinement statistics Values in parentheses are for the highest resolution shell. Wt-*Cb*FDH-CT, cryogenic temperature structure; Wt-*Cb*FDH-AT, ambient temperature structure.

	Wt-*Cb*FDH-CT	Wt-*Cb*FDH-AT	Val120Thr-*Cb*FDH (cryogenic)
PDB code	8hty	8iq7	8ivj
Data collection			
X-ray source	Turkish DeLight	Turkish DeLight	Turkish DeLight
Wavelength (Å)	1.54	1.54	1.54
Temperature (K)	100	298	100
Space group	*P*1	*P*1	*P*1
*a*, *b*, *c* (Å)	54.1, 69.0, 109.8	54.7, 69.5, 110.8	53.7, 68.6, 109.4
α, β, γ (°)	78.1, 89.5, 81.1	77.9, 89.1, 81.2	78.0, 89.6, 81.4
Resolution (Å)	24.75–1.39 (1.51–1.39)	30.73–2.10 (2.15–2.10)	25.42–1.90 (1.94–1.90)
CC_1/2_	0.999 (0.340)	0.916 (0.196)	0.622 (0.535)
CC*	1.000 (0.708)	0.978 (0.372)	0.876 (0.835)
〈*I*/σ(*I*)〉	11.74 (0.73)	7.90 (0.60)	3.00 (0.43)
Completeness (%)	98.82 (86.0)	85.90 (77.30)	99.7 (99.5)
Multiplicity	151.46 (148.36)	7.50 (3.30)	7.30 (4.60)
Refinement			
Resolution (Å)	21.83–1.40 (1.43–1.40)	30.70–2.10 (2.15–2.10)	24.30–1.90 (1.95–1.90)
No. of reflections	301717 (29123)	67181 (4741)	96104 (7897)
*R*_work_/*R*_free_	0.167/0.190 (0.380/0.372)	0.2433/0.2716 (0.4364/0.4810)	0.2469/0.2712 (0.4052/0.4226)
No. of atoms			
Protein	22225	22154	22275
Ligand/ion/water	2249	393	1459
*B* factors (Å^2^)			
Protein	22.94	18.89	22.47
Ligand/ion/water	35.07	17.02	26.27
Coordinate errors	0.18	0.38	0.30
R.m.s. deviations			
Bond lengths (Å)	0.015	0.002	0.002
Bond angles (°)	1.422	0.545	0.568
Ramachandran plot			
Favored (%)	97.40	97.66	98.02
Allowed (%)	2.60	2.34	1.98
Disallowed (%)	—	—	—

**Table 3:** Kinetic assay results for wild-type and Val120Thr *Cb*FDH

	Wild type	Val120Thr
*K*_m_ (m*M*)	2.00	1.30
*k*_cat_ (s^−1^)	9.48 × 10^2^	6.23 × 10^3^
*k*_cat_/*K*_m_ (*M* ^−1^ s^−1^)	4.74 × 10^2^	4.79 × 10^3^

## References

[bb13] Aronson, J. K. (2016). Editor. *Meyler’s Side Effects of Drugs*, 16th ed., pp. 437–443. Amsterdam: Elsevier.

[bb2] Atalay, N., Akcan, E. K., Gül, M., Ayan, E., Destan, E., Ertem, F. B., Tokay, N., Çakilkaya, B., Nergiz, Z., Karakadioğlu, G., Kepceoğlu, A., Yapici, I., Tosun, B., Baldir, N., Yildirim, G., Johnson, J. A., Güven, Ö., Shafiei, A., Arslan, N. E., Yilmaz, M., Kulakman, C., Paydos, S. S., Çinal, Z. S., Şabanoğlu, K., Pazarçeviren, A., Yilmaz, A., Canbay, B., Aşci, B., Kartal, E., Tavli, S., Çaliseki, M., Göç, G., Mermer, A., Yeşilay, G., Altuntaş, S., Tateishi, H., Otsuka, M., Fujita, M., Tekin, Ş., Çiftçi, H., Durdaği, S., Doğanay, G. D., Karaca, E., Türköz, B. K., Kabasakal, B. V., Kati, A. & DeMirci, H. (2023). *Turk. J. Biol.* **47**, 1–13.

[bb3] Brünger, A. T. (1992). *Nature*, **355**, 472–475. 10.1038/355472a0 18481394

[bb4] Brünger, A. T., Adams, P. D., Clore, G. M., DeLano, W. L., Gros, P., Grosse-Kunstleve, R. W., Jiang, J.-S., Kuszewski, J., Nilges, M., Pannu, N. S., Read, R. J., Rice, L. M., Simonson, T. & Warren, G. L. (1998). *Acta Cryst.* D**54**, 905–921. 10.1107/s0907444998003254 9757107

[bb5] Bulut, H., Yuksel, B., Gul, M., Eren, M., Karatas, E., Kara, N., Yilmazer, B., Kocyigit, A., Labrou, N. E. & Binay, B. (2021). *Appl. Biochem. Biotechnol.* **193**, 363–376. 10.1007/s12010-020-03429-0 32974869

[bb6] Calzadiaz-Ramirez, L. & Meyer, A. S. (2022). *Curr. Opin. Biotechnol.* **73**, 95–100. 10.1016/j.copbio.2021.07.011 34348217

[bb7] Chen, G.-Q. & Jiang, X.-R. (2018). *Curr. Opin. Biotechnol.* **50**, 94–100. 10.1016/j.copbio.2017.11.016 29223022

[bb8] Choe, H., Joo, J. C., Cho, D. H., Kim, M. H., Lee, S. H., Jung, K. D. & Kim, Y. H. (2014). *PLoS One*, **9**, e103111. 10.1371/journal.pone.0103111 PMC4111417 25061666

[bb9] Dumorné, K., Córdova, D. C., Astorga-Eló, M. & Renganathan, P. (2017). *J. Microbiol. Biotechnol.* **27**, 649–659. 10.4014/jmb.1611.11006 28104900

[bb10] Emsley, P., Lohkamp, B., Scott, W. G. & Cowtan, K. (2010). *Acta Cryst.* D**66**, 486–501. 10.1107/S0907444910007493 PMC2852313 20383002

[bb11] Eppinger, J. & Huang, K.-W. (2017). *ACS Energy Lett.* **2**, 188–195.

[bb12] Fischer, M. (2021). *Q. Rev. Biophys.* **54**, e1. 10.1017/S0033583520000128 33413726

[bb14] Fraser, J. S., van den Bedem, H., Samelson, A. J., Lang, P. T., Holton, J. M., Echols, N. & Alber, T. (2011). *Proc. Natl Acad. Sci. USA*, **108**, 16247–16252. 10.1073/pnas.1111325108 PMC3182744 21918110

[bb15] Garman, E. F. & Owen, R. L. (2006). *Acta Cryst.* D**62**, 32–47. 10.1107/S0907444905034207 16369092

[bb16] Gul, M., Ayan, E., Destan, E., Johnson, J. A., Shafiei, A., Kepceoğlu, A., Yilmaz, M., Ertem, F. B., Yapici, İ., Tosun, B., Baldir, N., Tokay, N., Nergiz, Z., Karakadioğlu, G., Paydos, S. S., Kulakman, C., Ferah, C. K., Güven, Ö., Atalay, N., Akcan, E. K., Cetinok, H., Arslan, N. E., Şabanoğlu, K., Aşci, B., Tavli, S., Gümüsboğa, H., Altuntaş, S., Otsuka, M., Fujita, M., Tekin, Ş., Çiftçi, H., Durdaği, S., Karaca, E., Türköz, B. K., Kabasakal, B. V., Kati, A. & DeMirci, H. (2023). *Sci. Rep.* **13**, 8123. 10.1038/s41598-023-33989-0 PMC10198979 37208392

[bb17] Guo, Q., Gakhar, L., Wickersham, K., Francis, K., Vardi-Kilshtain, A., Major, D. T., Cheatum, C. M. & Kohen, A. (2016). *Biochemistry*, **55**, 2760–2771. 10.1021/acs.biochem.6b00181 PMC4917879 27100912

[bb18] Hodel, A., Kim, S.-H. & Brünger, A. T. (1992). *Acta Cryst.* A**48**, 851–858.

[bb19] Jiang, W., Lin, P., Yang, R. & Fang, B. (2016). *Appl. Microbiol. Biotechnol.* **100**, 8425–8437. 10.1007/s00253-016-7613-6 27198726

[bb80] Li, C., Zhang, R., Wang, J., Wilson, L. M. & Yan, Y. (2020). *Trends Biotechnol.* **38**, 729–744. 10.1016/j.tibtech.2019.12.008 PMC7274900 31954530

[bb1] Liebschner, D., Afonine, P. V., Baker, M. L., Bunkóczi, G., Chen, V. B., Croll, T. I., Hintze, B., Hung, L.-W., Jain, S., McCoy, A. J., Moriarty, N. W., Oeffner, R. D., Poon, B. K., Prisant, M. G., Read, R. J., Richardson, J. S., Richardson, D. C., Sammito, M. D., Sobolev, O. V., Stockwell, D. H., Terwilliger, T. C., Urzhumtsev, A. G., Videau, L. L., Williams, C. J. & Adams, P. D. (2019). *Acta Cryst.* D**75**, 861–877.

[bb20] McCoy, A. J., Grosse-Kunstleve, R. W., Adams, P. D., Winn, M. D., Storoni, L. C. & Read, R. J. (2007). *J. Appl. Cryst.* **40**, 658–674. 10.1107/S0021889807021206 PMC2483472 19461840

[bb21] Miller, M., Robinson, W. E., Oliveira, A. R., Heidary, N., Kornienko, N., Warnan, J., Pereira, I. A. C. & Reisner, E. (2019). *Angew. Chem. Int. Ed.* **58**, 4601–4605. 10.1002/anie.201814419 PMC6563039 30724432

[bb22] Modarres, H. P., Mofrad, M. R. & Sanati-Nezhad, A. (2016). *RSC Adv.* **6**, 115252–115270.

[bb23] Murashima, K., Yoneda, H., Sumi, H. & Amao, Y. (2022). *New J. Chem.* **46**, 10004–10011.

[bb24] Nakasako, M. (2004). *Philos. Trans. R. Soc. London B*, **359**, 1191–1206. 10.1098/rstb.2004.1498 PMC1693410 15306376

[bb25] Nielsen, C. F., Lange, L. & Meyer, A. S. (2019). *Biotechnol. Adv.* **37**, 107408. 10.1016/j.biotechadv.2019.06.007 31200015

[bb26] Niks, D. & Hille, R. (2018). *Methods Enzymol.* **613**, 277–295. 10.1016/bs.mie.2018.10.013 30509470

[bb27] Popov, V. O. & Lamzin, V. S. (1994). *Biochem. J.* **301**, 625–643. 10.1042/bj3010625 PMC1137035 8053888

[bb28] Powers, P. O. (1953). *Ind. Eng. Chem.* **45**, 1063–1066.

[bb29] Schuler, E., Demetriou, M., Shiju, N. R. & Gruter, G. M. (2021). *ChemSusChem*, **14**, 3636–3664. 10.1002/cssc.202101272 PMC8519076 34324259

[bb30] Ye, J.-W., Lin, Y.-N., Yi, X.-Q., Yu, Z.-X., Liu, X. & Chen, G.-Q. (2023). *Trends Biotechnol.* **41**, 342–357. 10.1016/j.tibtech.2022.11.010 36535816

